# The Mechanism of Insulin-Like Growth Factor II mRNA-Binging Protein 3 Induce Decidualization and Maternal-Fetal Interface Cross Talk by TGF-β1 in Recurrent Spontaneous Abortion

**DOI:** 10.3389/fcell.2022.862180

**Published:** 2022-04-08

**Authors:** Rong-hui Zhu, Fang-fang Dai, Dong-yong Yang, Shi-yi Liu, Ya-jing Zheng, Ma-li Wu, Zhi-min Deng, Zi-tao Wang, Yu-wei Zhang, Wei Tan, Zhi-dian Li, Juan He, Xiao Yang, Min Hu, Yan-xiang Cheng

**Affiliations:** ^1^ Department of Obstetrics and Gynecology, Renmin Hospital of Wuhan University, Wuhan, China; ^2^ Department of Obstetrics and Gynecology, Peking University People’s Hospital, Beijing, China

**Keywords:** recurrent spontaneous abortion, decidualization, IGF2BP3, TGF-β1, maternal–fetal interface cross talk

## Abstract

Recurrent spontaneous abortion (RSA) is defined as the loss of two or more consecutive intrauterine pregnancies that are clinically established early in pregnancy. To date, the etiology and underlying mechanisms of RSA remain unclear. It is widely thought that the impairment of decidualization is inclined to induce subsequent pregnancy failure and leads to the dysregulation of extra-villous trophoblast invasion and proliferation through maternal–fetal cross talk. However, the mechanism of decidualization in RSA has yet to be understood. In our study, we demonstrate that decidual samples from RSA patients have significantly higher insulin-like growth factor 2 mRNA-binding protein 3 (IGF2BP3) and lower TGF-β1 levels compared to healthy controls. In addition, the overexpression of IGF2BP3 in human endometrial stromal cells (hESCs) can lead to the impairment of decidualization *in vitro*-induced model and the abnormal cell cycle regulation. Furthermore, TGF-β1 and MMP9 levels were greatly increased after decidualization, whereas IGF2BP3 overexpression inhibited endometrial mesenchymal decidualization by downregulating TGF-β1, impeding maternal–fetal interface cytokine cross talk, and limiting the ability of trophoblast invasion. In conclusion, our investigation first demonstrates that abnormal elevation of IGF2BP3 in the pregnant endometrium leads to the impairment of decidualization and abnormal trophoblast invasion, thereby predisposing individuals to RSA.

## Introduction

Recurrent spontaneous abortion (RSA) is defined as two or more consecutive pregnancy losses within 20–24 weeks of gestation with the same partner (Dimitriadis et al., 2020). RSA is a complex complication of pregnancy, and repeated embryo loss may lead to endometrial damage, endometritis, pelvic inflammatory disease, and infertility, posing a serious threaten to female reproductive health (Alves and Rapp, 2021). The etiology of RSA is complex, and mounting studies have shown that it is closely related to genetic abnormalities, anatomical abnormalities, endocrine disorders, metabolic dysfunction, autoimmune disorders, thrombotic tendencies, and reproductive tract infections (Schreiber et al., 2018; Carp, 2019; Colley et al., 2019).

Decidualization is stimulated by progesterone and cyclic adenosine monophosphate (c-AMP). In this process, the blastocyst adheres to the endometrial epithelium, the human endometrial stromal cells (hESCs) around the blastocyst are differentiated into round epithelial-like cells, that is, specialized secretory decidual cells (Gellersen and Brosens, 2014). Meanwhile, the orderly proliferation and apoptosis of hESCs are proceeded, accompanied by the formation of polyploid cells and neovascularization (Ng et al., 2020). This morphological differentiation and high-metabolic state make hESCs ready for subsequent embryo implantation. The highly expressed insulin-like growth factor binding protein-1 (IGFBP1) and prolactin (PRL) by decidualized hESCs can be used as marker molecules for the progression of decidualization (Kautz et al., 2015). More importantly, the decidualization provides important nutrients and immune environment for the maintenance of early pregnancy and placenta formation. The estrogen and progesterone, prostaglandins, transcription factors, cell cycle, signaling pathways, and many genes all play a role in the induction, growth, and degeneration of the decidua (Xiang et al., 2015; Shukla et al., 2019; Diniz-da-Costa et al., 2021). The establishment and maintenance of decidualization are critical for embryo implantation and pregnancy establishment and maintenance. The impairment of hESCs decidualization leads to dysfunction decidual maturation, which is closely associated with abnormal tolerance at the maternal–fetal interface and RSA. In-depth investigation of the factors affecting this biological behavior of endometrial interstitial cell metaplasia can help to elucidate the pathogenesis of RSA and provide an important basis for finding more effective and precise treatments.

Insulin-like growth factor 2 (IGF2), a multifunctional cell proliferation regulator, has an important role in promoting the differentiation and proliferation of embryonic and tumor cells (Chao and D’Amore, 2008; Masunaga et al., 2020). rRNA-binding protein (RBP) is an important checkpoint for gene expression at the RNA level and mediates posttranscriptional regulation of gene expression by interacting with mRNA (Köster et al., 2017). Insulin-like growth factor 2 mRNA-binding protein 3 (IGF2BP3), namely, IMP3, as an RBP oncoprotein, can bind to the 5-untranslated region of IGF2-expressing mRNAs and activates transcriptional targets (Schneider et al., 2019), subsequently maintaining mRNA stability and regulating their transcriptional expression (Ren et al., 2020). Studies have reported that IGF2BP3 has different targets of action in tumor growth, stem cell proliferation, and other systems, and mainly function in promoting cell differentiation, proliferation, and invasion (Ennajdaoui et al., 2016; Yin et al., 2020). At present, it is unclear that whether IGF2BP3 involves in the impairment of hESCs decidualization in RSA. An experiment showed that IGF2BP3 is localized in the decidual endometrium during early gestation (Zheng et al., 2008).

The transforming growth factor *β* (TGF-β)-signaling pathway plays an important role in cell differentiation, and IGF2 regulates the transcription of TGF-β-related pathway molecules in the epigenetic of tumor cells (Chen and Gingold, 2020). Evidence showed that the dysfunction of TGF-β1 can lead to adverse pregnancy complications, such as RSA, pre-eclampsia, fetal growth restriction, and reduced fertility (Hadinedoushan et al., 2015; Alkhuriji et al., 2020). On the one hand, the dysfunction of TGF-β1 is associated with the dysfunction of hESCs decidualization (Ni and Li, 2017); on the other hand, as we previously reviewed, TGF-β1 can promote biological behaviors such as trophoblast invasion and proliferation by regulating epithelial–mesenchymal transition (EMT) (Li et al., 2021a).

Here, we found that decidual samples from RSA patients have significantly higher insulin-like growth factor 2 mRNA-binding protein 3 (IGF2BP3) and lower TGF-β1 levels compared to healthy controls. Moreover, the overexpression of IGF2BP3 in human endometrial stromal cells (hESCs) can lead to the impairment of decidualization *in vitro*-induced model and the abnormal cell cycle regulation. Furthermore, TGF-β1 and MMP9 levels were greatly increased after decidualization, whereas IGF2BP3 overexpression-inhibited endometrial mesenchymal decidualization by downregulating TGF-β1, impeding maternal–fetal interface cytokine cross talk, and limiting the ability of trophoblast invasion. Finally, we successfully established the RSA animal model and found that the TGF-β1 level in serum of RSA is lower than that of the normal pregnant mouse. Our investigation may contribute to reveal the special mechanism of the impairment of decidualization and maternal–fetal interface cross talk dysfunction in RSA.

## Materials and Methods

### Decidual Tissue Collection

This study included 12 patients with recurrent spontaneous abortion (RSA) between the ages of 20 and 35 (mean age 28.2 ± 3.4 years) who were treated at Wuhan University’s Renmin Hospital between 2020 and 2021. Healthy controls were 12 women aged 20–35 (mean age 27.7 ± 2.1 years) who had healthy early pregnancies that were intentionally terminated for non-medical reasons (HC). The gestational ages of the HC and RSA groups when the pregnancy was terminated were 9.2 ± 1.3 weeks and 8.5 ± 2.5 weeks, respectively. The following were the exclusion criteria (Dimitriadis et al., 2020): uterine deformity discovered by a pelvic exam and ultrasound (Alves and Rapp, 2021), chromosome abnormalities (Colley et al., 2019), endocrine or metabolic diseases (Carp, 2019), infection (Schreiber et al., 2018), and other recognized reasons. The Renmin Hospital Research and Ethics Committee approved the form and use of the decidual tissues (WDRY2020-K218). Before collecting decidual tissues, all subjects gave their written agreement. After suction and curettage, the decidual tissues were collected and washed with PBS to eliminate blood. Before being used, the tissues were immediately frozen in liquid nitrogen and then preserved at −80°C.

### Cell Culture and Treatment

The human choriocarcinoma cell lines HTR8/SVneo and the immortalized human endometrial stromal cells (hESCs) were donated by Reproductive Center of Sun Yat-sen University. The cells were cultured in Dulbecco’s modified Eagle’s medium (DMEM)/F-12 (Meilunbio, China) supplemented with 10% fetal bovine serum (FBS) (Gibco, Life Technologies, Grand Island, NY), 100 units/mL of penicillin and streptomycin (Invitrogen, Waltham, MA, United States). hESCs were cultured in phenol red-free Dulbecco’s modified Eagle’s medium (DMEM)/F-12 (Meilunbio, China) plus 10% FBS at 37°C in a 5% CO_2_ atmosphere. hESCs were treated with 1 mol/L medroxyprogesterone-17-acetate (MPA) (MedChemExpress, China) and 0.5 mmol/L N6,20-O-dibutyryladenosine cAMP sodium salt (db-cAMP) (MedChemExpress, China) to promote decidualization *in vitro*. The medium was replaced every 2 days. Extrinsic TGF-β1 was obtained from recombinant proteins (MCE, United States) and added at a concentration of 10 ng/ml.

### Lentivirus Infection

Overexpression plasmid and small RNA interference (siRNA) encapsulated by stable knockdown (shRNA) lentivirus were used in this study. Double-stranded human IGF2BP3 gene shRNAs and overexpression plasmid were purchased from Hanheng biology (Shanghai, China). The optimal amount of virus infection was performed according to the manufacturer’s instructions. The MOI = 30 was chosen to conduct the subsequent experiments. The transfected cells were cultured in DMEM/F12 containing 10% FBS. After 48 h of first infection, the stable infected cells were selected with 5 μg/ml of puromycin (Invitrogen, United States). Overexpression and knockdown efficiency were verified by qPCR or Western blotting.

### Real-Quantitative Polymerase Chain Reaction Analysis

Total RNA was extracted from cells or tissues by TRIzol reagent (Invitrogen) according to the manufacturer’s instructions. A spectrophotometer (NanoDrop 2000c; Thermo Fisher Scientific, Waltham, MA, United States) was used to determine the concentration and purity of RNA. Total RNA (1 μg) was reverse transcribed using a Reverse Transcription kit (Yeasen Biotechnology Co. Ltd.), and the resultant cDNA was utilized as the qPCR template. Gene expression quantification was carried out in triplicate on a Bio-Rad PCR system in an Applied Biosystems CFX96TM Real-Time PCR system using SYBR GREEN PCR Master Mix according to the real-time PCR manufacturer’s instructions (Shanghai Yisheng Co., Ltd.). To standardize gene expression levels, the PCR products were measured using the 2^−ΔΔCt^ technique relative to glyceraldehyde-3-phosphate dehydrogenase (GAPDH). Supplementary Table lists the primers that are utilized.

### Western Blot Analysis

Total protein extracts were produced using RIPA buffer including PMSF protease inhibitors (Beyotime Biotechnology, China) and a phosphatase inhibitor (Beyotime Biotechnology, China) from homogenized tissues or cultured cells. An Enhanced BCA Protein Assay Kit was used to measure protein (Beyotime Biotechnology, China). SDS-PAGE was used to separate equal quantities of protein before wet transfer onto 0.45 mm/0.22 mm polyvinylidene difluoride (Merck Millipore, Billerica, MA). The membranes were blocked for 2 h at room temperature with 5% non-fat milk. After blocking, the membranes were incubated with primary antibodies against IGF2BP3 (1:1000; ABclonal, China, Cat: #A6099), PRL (1:1,000; ABclonal, China, Cat: #A1618), IGFBP1 (1:1,000; cst, Cat: #31025S), TGFb1 (1:1,000; ABclonal, China, Cat: #A15103), or GAPDH (ABclonal, China, Cat: #AC036) overnight at 4°C. Then, the secondary antibodies were used (1:5,000; ABclonal, China, Cat: #ASO14) to incubate the membranes for 1 h at room temperature.

The protein expression was detected by the chemiluminescent detection system (Bio-Rad, United States) once adding the ECL Enhanced Plus Kits (RM0021P, ABclonal, China). Protein expression was analyzed using Image Lab Analyzer software.

### Immunohistochemistry

Immunohistochemical staining was performed on paraffin-embedded tissue sections using mouse and rabbit specific HRP/DAB (ABC) detection IHC kits (ab64264; Abcam, Cambridge, United Kingdom). Briefly, tissue sections were deparaffinized and rehydrated. Ethylenediaminetetraacetic acid (EDTA) was used for epitope retrieval. Sections were blocked with BSA and incubated with primary antibodies overnight at 4°C (the antibody against IGF2BP3 used for immunohistochemistry was the same as that used for Western blotting) was followed by HRP-conjugated secondary antibodies. Tissues were subsequently counterstained with hematoxylin and diaminobenzidine and hydrated. PBS was substituted for the primary antibody as a negative control. The positive staining intensity and relative protein expression were evaluated by ImageJ Pro Plus version 6.0 software (Media Cybernetics Inc.,).

### Flow Cytometrical Determination of Cell Cycle

For the flow cytometrical analysis of cell cycle, cells were trypsinizated, collected by centrifugation, fixed by 70% ethanol at 4°C overnight, centrifuged again, and incubated at 37°C in a buffer containing 0.5 μg/mL RNase A and 0.1 mg/mL propidium iodide for 30 min. Samples were analyzed by BD FACSCalibur^TM^, and data were analyzed using FlowJo10.0 software.

### Transforming Growth Factor Beta 1 Cytokine Assay

With human TGF-β1 ELISA kits (ELK1185, Wuhan, China), concentrations in the conditioned medium of infected hESCs were measured, and the level of TGF-β1 in animal serum were detected by the mouse TGF-β1 ELISA kits (ELK1186, Wuhan, China). Analyses were performed according to the manufacturers’ instruction for ELISA kit. On a microplate reader (Thermo Fisher Scientific Inc., United States), the optical density of each well at 450 nm was measured, and concentrations were calculated using standard curves. The concentration (pg/mL) of the results is compared to the control.

### Transwell Assays

The invasion of trophoblast cells across Matrigel (40 μL; dilution 1:8; Sigma, St Louis, MO) was evaluated using Matrigel pre-coated Transwell inserts with 8.0‐nm diameter (Corning, Cambridge, MA), and the migration of trophoblasts was assessed using Matrigel-free pre-coated Transwell inserts. hESCs that had been transfected with OV-NC, OV-IGF2BP3 ,or sh-IGF2BP3 were seeded into the lower chamber. MPa and dbcAMP were used the following day to induce decidualization, and experimental groups started other drug treatments after 48 h of induction. After 3 days, HTR8/SVneo trophoblast cells were planted into the top chamber of 24-well plates that had been pre-coated with 40 μl 1:8 diluted Matrigel. HTR8/SVneo cells were cultured in basal medium, whereas hESCs induced by decidualization were placed in 20% FBS media in the lower chamber. Co-cultured cells studied for migration and invasion were incubated for 24 or 48 h, respectively. The inserts were then removed and rinsed in PBS, and the non-invading cells and Matrigel were wiped off the filter’s top surface using a cotton bud. The inserts were then dyed with crystalline iodine after being fixed in methanol for 10 min at room temperature. Under a fluorescent microscope (Olympus, Japan), the cells that migrated to the lower surface were viewed and counted in five randomized areas at a magnification of ×200. Each experiment was conducted out in triplicate and repeated three times.

### Scratch Wound-Healing Assay

HTR8/SVneo cells/well (2 × 10^5^) (three replicates per group) were seeded into a 12-well plate for the scratch wound assay and incubated until confluent. The monolayer was scraped with a tip and washed with serum-free medium to remove detached cells. Then, the cells were cultured in the pre-collected hESCs cell culture supernatant or in the complete medium supplemented with TGF-β1 (10 nM) or without special treatment.

With the fluorescence microscope, photographs of the wound area were taken at 0, 24, and 48 h. ImageJ software version 1.51 was used to quantify the data by assessing the portions of the scratch that were not covered by cells. The closure rate was measured as a percentage of the area at 0 h. Migration area (%) = (initial wound area—remaining area of the wound at the point of measurement)/initial wound area × 100.

### Spontaneous Abortion Animal Model Construction

Twenty CBA/J female mice and five BALB/c male mice and five DBA/2 male mice (6-week-old) were purchased from Beijing Vital River Laboratory Animal Technology Co. Ltd., China. They were in adaptive culture for 5 days in specific pathogen-free (SPF) in Renmin Hospital of Wuhan University. Ten CBA/J female mice and five BALB/c male micee were mated to construct the control model, ten CBA/J female micee and five BALB/c male micee were mated to construct the normal pregnancy control model, and ten CBA/J female mice and five DBA/2 male mice were mated to construct the spontaneous abortion model (Clark, 2020). Detection of a vaginal plug was chosen to indicate day 0.5 of gestation. All mice were killed at day 12.5 to examine and calculate the embryo resorption rate. All animal studies were approved by the ethics committee for laboratory animal welfare (IACUC) of Renmin Hospital of Wuhan University [No. WDRM animal (f) No. 20201207].

### Statistical Analysis

Each experiment was independently repeated at least three times. All data were analyzed using GraphPad Prism statistical software (Version 6.0). Differences between two groups were analyzed by Student’s *t-*test. All data are presented as the mean ± SEM. At *p* < 0.05, differences were judged statistically significant.

## Results

### IGF2BP3 Expression is Increased in the Decidual Tissues of Recurrent Spontaneous Abortion Patients

**FIGURE 1:**
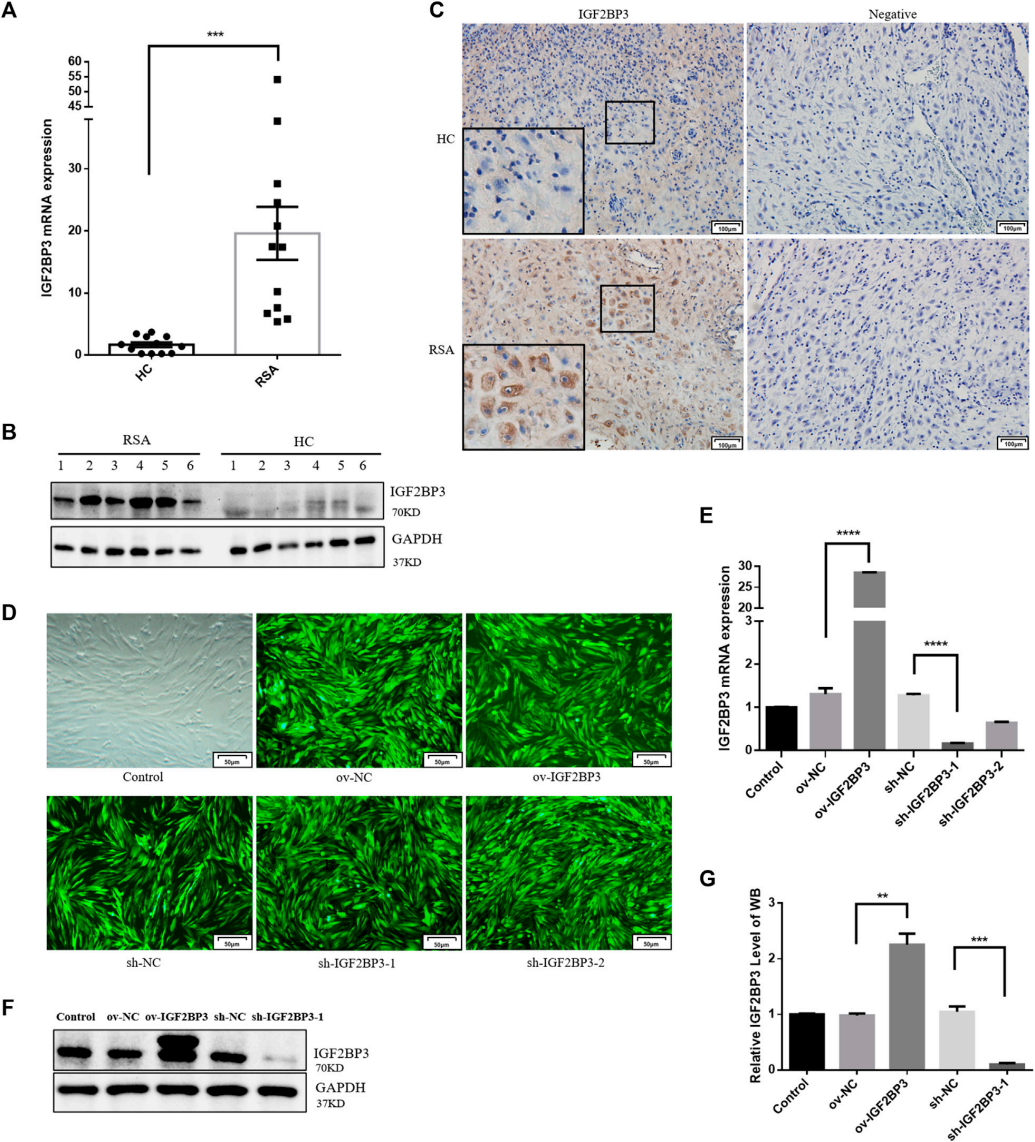
IGF2BP3 in decidual tissues and constructing cell models **(A)** IGF2BP3 mRNA levels in the decidual tissues of RSA patients and HCs (*n* = 12 for each group) were measured by qPCR. The relative RNA amount was calculated using the 2^−ΔΔCt^ method and normalized to internal control GAPDH, **(B)** IGF2BP3 protein levels relative to GAPDH were determined by Western blot (*n* = 6 for each group), **(C)** immunohistochemical staining showed the localization and relative quantification of IGF2BP3 in recurrent spontaneous abortion (RSA) and normal pregnancy (HC) decidual tissues. Brown staining represents the target protein. Inserts show a higher magnification of the square area. Scale bar = 100 μm, **(D)** fluorescence expression of hESC cells infected with lentivirus. Scale bars = 50 μm, **(E)** qRT-PCR assays of relative IGF2BP3 mRNA expression showing its overexpression and knockdown in hESC cells, and **(F,G)** Western blot assays of relative levels of IGF2BP3 protein showing its overexpression and knockdown in hESC cells. Each experiment was independently performed three times. ***p < 0.001 and ****p < 0.0001 vs. control. HC, healthy control pregnancy; RSA, recurrent spontaneous abortion.

We used qPCR and Western blot analyses of first trimester decidual tissues from RSA and healthy control (HC) women to see if IGF2BP3 plays a role in the etiology of RSA. The results show that IGF2BP3 expression was considerably higher in the decidual tissues of RSA patients than that of HCs at both the mRNA and protein levels (Figures 1A,B), thus indicating its possible role in the development and functional regulation of decidua. The immunohistochemical results further confirmed these findings. We prepared paraffin sections of decidual tissues of HC and RSA and performed immunohistochemical analysis using an antibody against IGF2BP3. IGF2BP3 was mainly localized within decidual cells in RSA decidual tissue compared to HCS, with elevated expression (Figure 1C), consistent with a role for this protein in RSA-influencing decidual function.

### Confirmation of IGF2BP3 Overexpression and Knockdown

To investigate the role of IGF2BP3 in decidualization *in vitro*, we infected hESC cells with lentiviruses-encoding overexpression or the knockdown IGF2BP3 gene (Figure 1D). IGF2BP3 overexpression and silencing were confirmed in whole cell lysates at the mRNA and protein levels (Figures 1E–G). The level of IGF2BP3 mRNA was 21.8-fold greater than that of control after infection with the overexpression vector (*p* < 0.001), but 12.4 percent of that in the control group following infection with the knockdown vector (*p* < 0.05). Protein levels were also 2.3-fold greater (*p* < 0.01) and 9.7% of control levels (*p* < 0.001) in cells from the overexpression and knockdown groups (Figures 1E–G).

### IGF2BP3 had No Special Changes During Decidualization *In Vitro*

**FIGURE 2:**
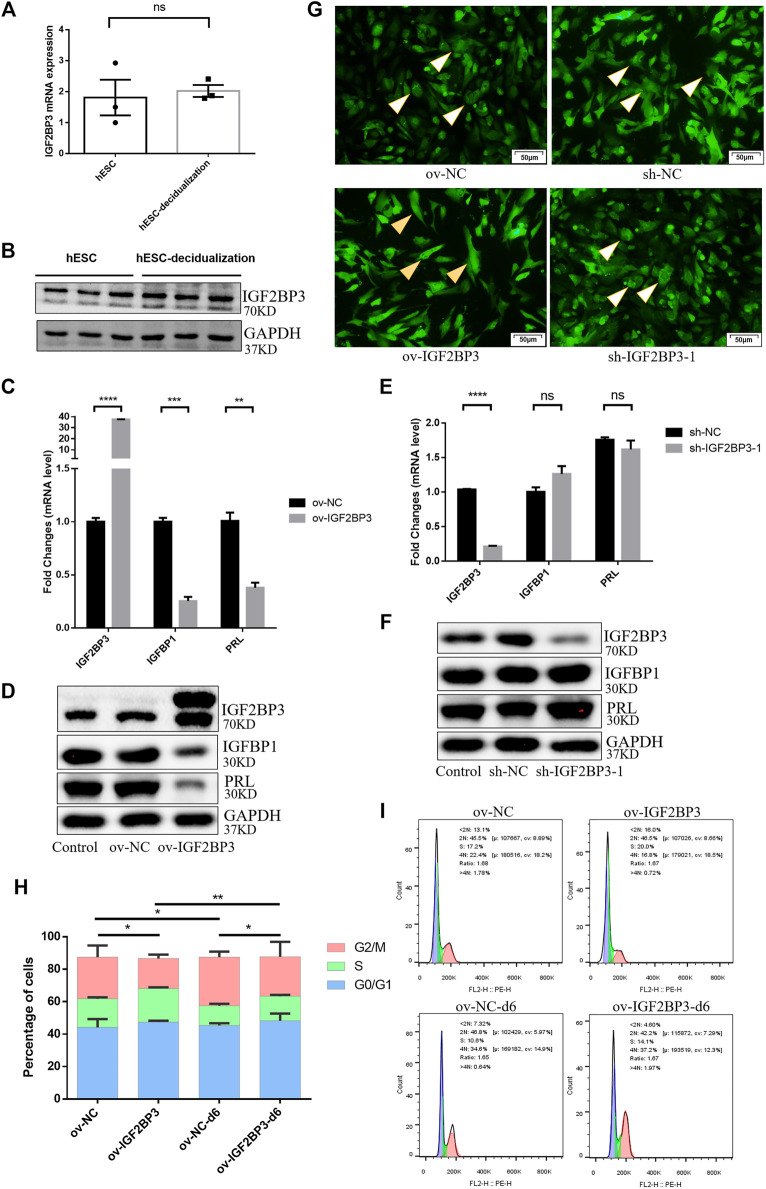
Role of IGF2BP3 in decidualization **(A,B)** Levels of IGF2BP3 mRNA and protein in hESCs were not significantly different with control cells after induction of decidualization (MPA and db-cAMP added for 6 days), **(C,D)** IGF2BP3 overexpress caused impaired decidualization of hESCs. The decidualization markers (IGFBP1 and PRL) reduced in HESCs upon induction of decidualization *in vitro* after infected with ov-IGF2BP3, **(E,F)** IGF2BP3 knockdown did not affect the decidualization of hESCs. After *in vitro* induction of decidualization and after infection with IGF2BP3-1, the decidualization markers (IGFBP1 and PRL) in hESCs were not significantly different compared with NC, **(G)** influence of IGF2BP3 knockdown or overexpress on the cell morphology of HESCs after decidualization. Decidualization was induced on hESCs infected with the respective NC, sh-IGF2BP3-1, or ov-IGF2BP3. Arrows point to the morphology of the four groups of hESCs at day 6. Scale bars = 50 μm, and **(H,I)** cell cycle distribution of ov-IGF2BP3 or ov-NC-infected hESC during decidualization *in vitro*. Cell cycle distribution in the process of the cells was analyzed after being cultured in DMEM/F12 containing 2% FBS (control group) or decidualization medium for 0 day and 6 days, respectively. Data represent mean ± SEM from three separate experiments, and were shown as the mean ± SEM. ns *p* ≥ 0.05 *p < 0.05, **p < 0.01, ***p < 0.001, and ****p < 0.0001 vs. control.

To investigate the involvement of IGF2BP3 in the pathogenesis of RSA, we used *in vitro* experiments of hESCs to confirm the functional role of IGF2BP3 during decidualization. *In vitro* experiments with hESCs were performed to establish that IGF2BP3 plays a functional role in the decidualization process. Therefore, we used db-cAMP and MPA to induce decidualization in hESCs for 6 days and found no significant differences in IGF2BP3 mRNA and protein expression compared to uninduced control cells. (Figures 2A,B).

### Overexpression of IGF2BP3 Attenuates Decidualization

To examine how IGF2BP3 impacts endometrial decidualization, we infected hESCs with sh-IGF2BP3 and ov-IGF2BP3 to induce decidualization with db-cAMP and MPA.

The results showed the mRNA levels of endometrial decidualization biomarkers (IGFBP1 and PRL) were significantly increased in the induced cells. In ov-IGF2BP3 cells, the levels of the decidualization markers IGFBP1 and PRL were greatly decreased (Figure 2C,D). Notably, IGF2BP3 knockdown had no effect on the levels of IGFBP1 and PRL (Figures 2E,F), which was consistent with the fact that normal induction of decidualization had no effect on the expression of IGF2BP3 (Figures 2A,B). We also noted that the overexpression of IGF2BP3 during decidualization resulted in differences in the morphogenesis of db-cAMP and MPA-induced hESCs, whereas on day 6 of induction control, cells were enlarged and spherical, the morphology of ov-IGF2BP3-transfected cells remained fibroblast-like (Figure 2G), clearly showing that the overexpression of IGF2BP3 inhibits the widely characterized morphological reprogramming of cells that happens during decidualization.

### Overexpression of IGF2BP3-Affected Cell Cycle Distribution During Human Endometrial Stromal Cells Decidualization

To investigate how IGF2BP3 inhibits cell decidualization, we performed cell cycle assays. hESCs-overexpressing IGF2BP3 or a scramble control was induced to be decidualized by progesterone and db-cAMP for 6 days, and differences in cell cycle distribution were detected by flow cytometry. A decrease in the percentage of cells in S-phase during decidualization was shown by comparing uninduced and decidualized day 6 hESCs (scramble control) (17.6 ± 0.91% in D0 and 12.2 ± 1.13% in D6). However, there was no significant difference in the proliferation rate (S + G2/M) during decidualization (43.23 ± 8.13% in d0 and 42.1 ± 2.28% in D6). The same trend was also obtained in the overexpression IGF2BP3 group including S-phase (20.7 ± 0.74% in D0 and 15.1 ± 0.81% in D6) and proliferation rate (39.13 ± 2.26% in D0 and 39.43 ± 8.45% in D6). (Figures 2H,I). It was shown that ESCs could not enter the S-phase during decidualization, resulting in cell cycle exit and differentiation into decidual cells that control embryo implantation. In addition, we also found that the upregulation of IGF2BP3 led to an accumulation in the S-phase cell phase. Interestingly, the overexpression of IGF2BP3 similarly led to an accumulation of S-phase cells in both cell groups at day 6 of induction, with a significant increase compared with controls (Figures 2H,I). The earlier results suggest that the overexpression of IGF2BP3 may lead to enhanced hESCs proliferative activity, failure to normally exit the cell cycle, and differentiate into decidual cells under induced decidualization conditions.

### IGF2BP3 Overexpression Mediates Transforming Growth Factor Beta 1-Signaling Pathway Inhibition Leads to Impaired Decidualization

**FIGURE 3:**
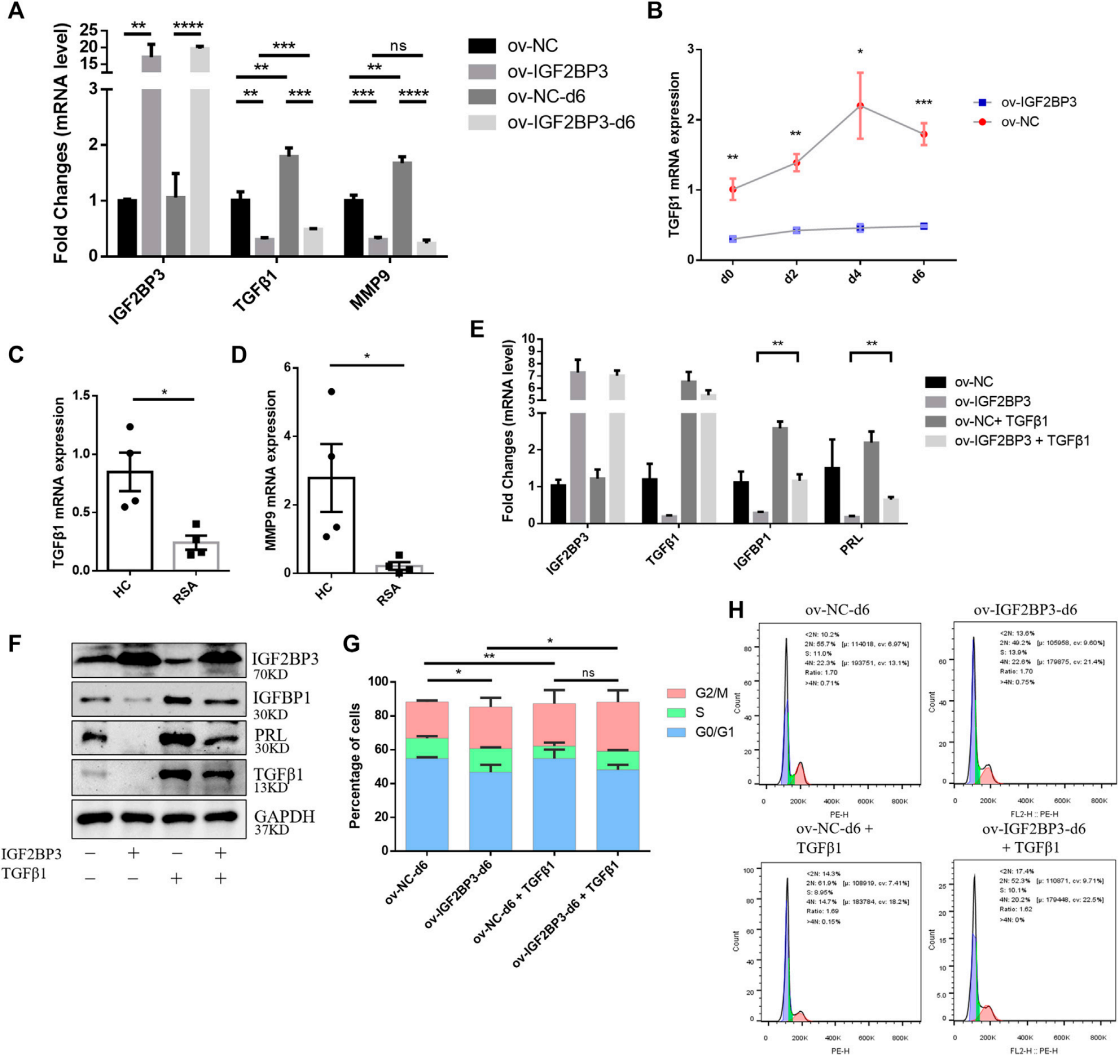
IGF2BP3 and TGF-β1 in decidualization **(A)** mRNA expression of decidualization-related transcriptional factors including TGF-β1 and MMP9 decreased in HESCs upon the induction of decidualization *in vitro* after infected with ov-IGF2BP3 **(B)** IGF2BP3 overexpression inhibits TGF-β1 time-dependent increase during the decidualization of hESCs, **(C,D)** TGF-β1 and MMP9 mRNA levels were reduced in decidua of RSA compared with HCs patients (*n* = 4 per group), **(E,F)** addition of exogenous TGF significantly increased the decidualization of hESCs and restored the RNA and protein expression levels of decidualization marker molecules (IGFBP1 and PRL) in the IGF2BP3 overexpression group, and **(G,H)** cell cycle distribution of ov-IGF2BP3 or ov-NC-infected hESC during decidualization *in vitro*. Addition of TGF-β1 to ov-IGF2BP3 or ov-NC-infected hESCs, comparison of cell cycle distribution during day 6 *in vitro* decidualization. TGF can reverse the S-phase accumulation caused by IGF2BP3 overexpression. Data represent mean ± SEM from three separate experiments, and are shown as the mean ± SEM. *p < 0.05, **p < 0.01, ***p < 0.001, and ****p < 0.0001 vs. control.

A previous study provided evidence showing that the downregulation of IGF2BP3 activates the TGF-β1/Smads axis (Chen et al., 2013). Subsequently, the downregulation of TGF-β1 can inhibit endometrial stromal cell decidualization (23). Because IGF2BP3 plays a critical role in decidualization, we investigated whether IGF2BP3 regulates the TGF-β1-signaling pathway during decidualization. We found that the mRNA expression levels of TGF-β1 and MMP9 in hESCs increase dramatically during decidualization. However, IGF2BP3 overexpression decreased TGF-β1 and MMP9 mRNA levels (Figure 3A), inhibited the increase of TGF-β1 in time-dependent decidualization (Figure 3B). In addition, the expression of TGF-β1 and MMP9 in the uterine decidua of RSA patients was significantly reduced compared to HC women (Figures 3C,D), which further confirmed the results of the *in vitro* cell experiments. The results are also in keeping with other reports (Toth et al., 2020; Lu et al., 2021).

Furthermore, we investigated whether IGF2BP3 overexpression-impaired decidualization in a TGF-β1-dependent manner. Extrinsic TGF-β1 was added to the induction decidualization medium to agonize its downstream pathways. TGF-β1 significantly promoted the decidualization of hESCs and restored the RNA and protein expression levels of decidualization marker molecules (IGFBP1 and PRL) in the IGF2BP3-overexpressing group compared with their respective controls (Figures 3E,F). Moreover, we further found that IGF2BP3 overexpression affects cell cycle distribution during hESCs decidualization, possibly through TGF-β1-signaling pathways (Figures 3G,H). By comparing cell cycle distribution in the TGF-β1-added group and the control group during the addition of metaphase, it showed that S-phase accumulation caused by IGF2BP3 overexpression can be reversed to promote stromal cell differentiation into metaphase cells, which control embryo implantation by inducing cell cycle exit.

### IGF2BP3 Overexpression Disrupts Cross Talk Between Decidual Stromal Cells and Trophoblast Cells

Finely tuned management of human trophoblast invasion at the maternal–fetal interface is critical for a successful pregnancy outcome. Specifically, impaired decidualization prevents endometrial stromal cells from developing into normal decidual cells, leads to abnormalities in trophoblast invasive capacity, and limits further trophoblast proliferation and differentiation (Sharma et al., 2016; Abbas et al., 2020). We first evaluate whether IGF2BP3 is engaged in the communication between decidual stromal cells and trophoblast cells in order to better understand any possible involvement for IGF2BP3 in the molecular mechanism underpinning the regulation of trophoblast invasion by decidua.

**FIGURE 4:**
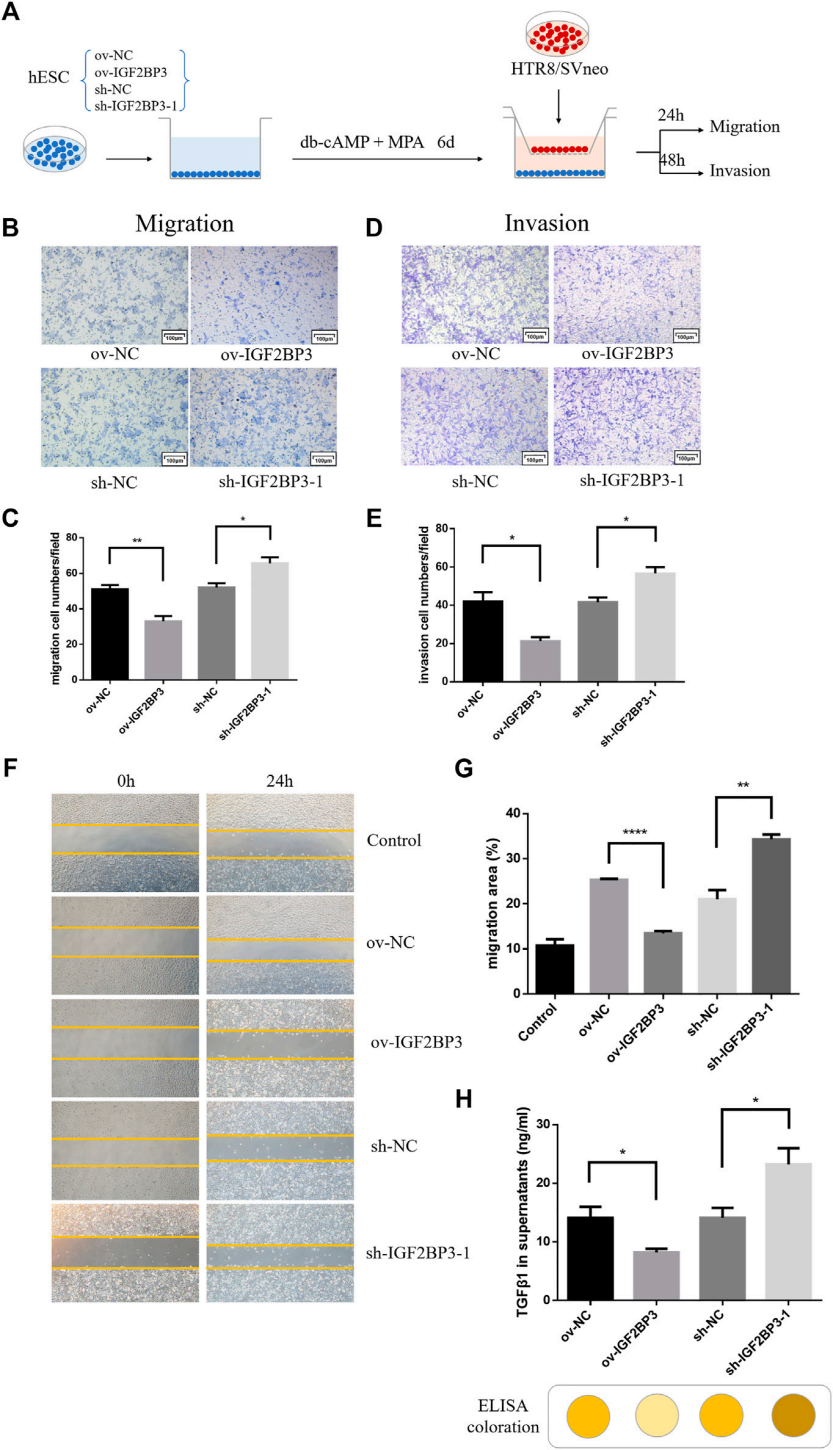
IGF2BP3 in maternal–fetal interface cells cross talk **(A)** schematic diagram of the process of co-culture experiments, **(B)** transwell migration of HTR8/SVneo cells in co-culture with IGF2BP3-transfected HESCs, **(C)** quantification statistics of the results of cell migration, **(D)** transwell invasion of HTR8/SVneo cells in co-culture with IGF2BP3-transfected HESCs **(E)** Quantification statistics of the results of cell invasion, **(F)** scratch wound-healing by different decidual cell culture supernatants for culturing trophoblasts, **(G)** HTR8/SVneo cells migration quantified by wound-healing index. Migration area (%) = (A0—An)/A0 × 100, and **(H)** ELISA measurement of the expression levels of TGF-β1 in decidual cell culture supernatants of sh-IGF2BP3, ov-IGF2BP3, and their respective control groups. Each experiment was independently performed three times. *p < 0.05, **p < 0.01, ***p < 0.001, and ****p < 0.0001 vs. control.

HESCs transfected with sh-IGF2BP3 and ov-IGF2BP3 and their scrambled controls were seeded in the bottom chamber. Conditioned medium was cleared 6 days after decidualization induction with db-cAMP and MPA. Subsequently, HTR8/SVneo trophoblast cells were implanted in the upper inserts and co-cultured with the IGF2BP3-infected decidual cell for 24–48 h (Figure 4A). These experiments revealed that co-culture with IGF2BP3 knockdown cells could effectively enhance trophoblast cell invasion and migration ability of trophoblast cells compared with normal decidual cells (Figures 4B–E). Conversely, co-culture with IGF2BP3 overexpression cells greatly inhibited the invasion and migration ability of trophoblast cells (Figures 4B–E).

To investigate in isolation, the effects of IGF2BP3-transfected cells on HTR8/SVneo cell migration *via* exocrine secretion, we collected different decidual cell culture supernatants for culturing trophoblasts for wound scratch assays. The results showed that all experimental groups exhibited better wound-healing ability compared with the blank control (Figures 4F,G). Among them, HTR8/SVneo cells cultured with the supernatant of sh-IGF2BP3 cells showed an increased ability to migrate compared with the scrambled control, whereas the supernatant of IGF2BP3-overexpressing cells inhibited HTR-8/Svneo cell migration (Figures 4F,G).

To investigate which cytokine production was specifically secreted by IGF2BP3-transfected cells and affected migration of HTR8/SVneo cells, we collected decidual cell culture supernatants of sh-IGF2BP3 and ov-IGF2BP3 and measured TGF-β1 expression levels in them using an ELISA kit. It displayed that the higher TGF-β1 in the supernatant of sh-IGF2BP3 cells compared with their respective scrambled controls (Figure 4H). However, TGF-β1 levels were significantly reduced in the supernatant of IGF2BP3-overexpressing cells (Figure 4H). We also found that the relative expression of TGF in the culture supernatant of uninduced hESCs was significantly lower than that of hESCs that had been induced to decidualize and was not statistically different from the blank control (Supplementary Figure S1A). This result further proved the conclusion of the earlier study.

### IGF2BP3 Utilizes Transforming Growth Factor Beta 1 Regulation of Cross talk Between Decidual Stromal Cells and Trophoblast Cells

**FIGURE 5:**
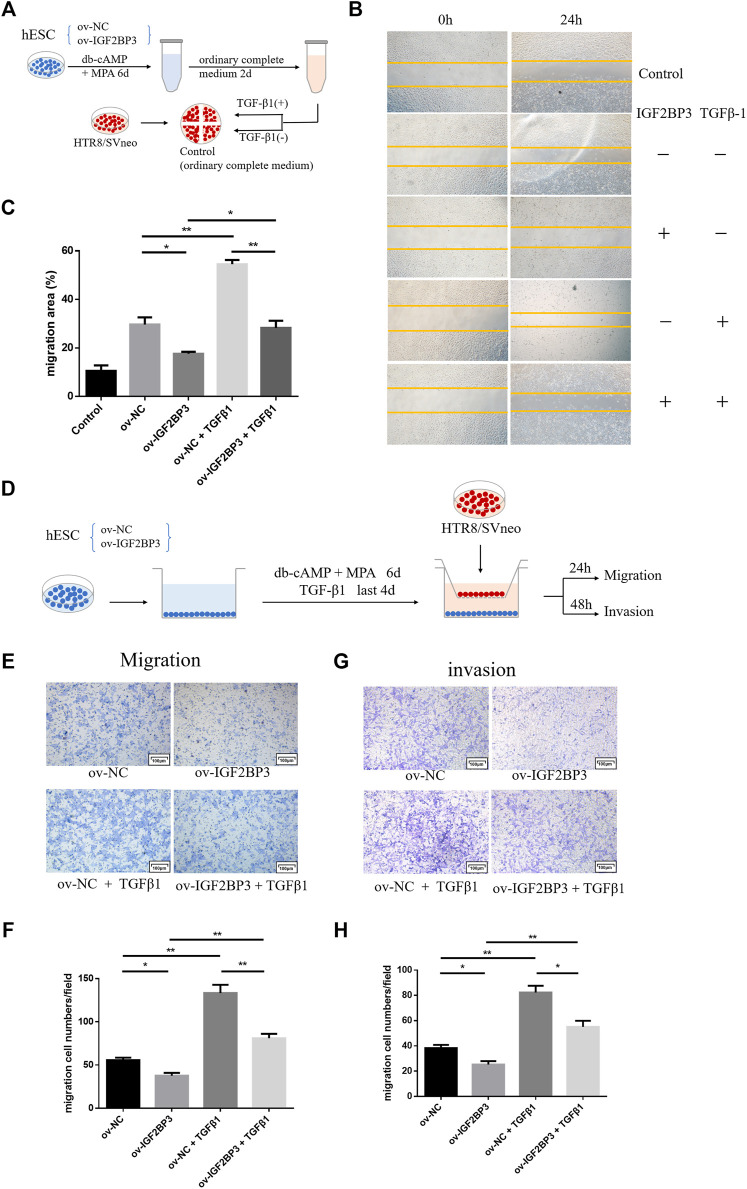
IGF2BP3 and TGF-β1 in the maternal–fetal interface cells cross talk **(A)** schematic diagram of the process of scratch wound-healing, **(B)** trophoblast cells were cultured for wound scratch assays using ov-IGF2BP3 and control decidual cells culture supernatants with selective TGF-β1 addition, **(C)** HTR8/SVneo cells migration quantified by wound-healing index. Migration area (%) = (A0—An)/A0 × 100, **(D)** schematic diagram of the process of co-culture and rescue experiments, **(E,F)** transwell migration and invasion of HTR8/SVneo cells co-cultured with IGF2BP3-transfected hESCs supplemented with TGF-β1, and **(G,H)** quantification statistics of the results of cell migration and invasion. Each experiment was independently performed three times. *p < 0.05, **p < 0.01, and ***p < 0.001 vs. control.

To explore whether ov-IGF2BP3-transfected decidual cells regulate trophoblast migration and invasion through TGF-β1 exocrine secretion, we collected different decidual cell culture supernatants and selectively added TGF-β1 activators for culturing trophoblast cells for the wound scratch assay (Figure 5A). The results showed that the exogenous addition of TGF-β enhanced the migration ability of cultured TR8/SVneo cells for all the supernatant experimental groups compared with the blank group. Relative inhibition of HTR8/SVneo cell migration by supernatants from IGF2BP3-overexpressing cells relative to scrambled controls remained, but could be partially reversed by TGF-β1 (Figures 5B,C).

In the co-culture group, we further verified that the overexpression of IGF2BP3 could inhibit trophoblast cell migration and invasion by regulating the TGF-β1 axis. TGF-β1 was added during the induction of decidualization of IGF2BP3 transfected hESCs in the lower chamber for 4 days, after the removal of conditioned medium, HTR8/SVneo trophoblast cells were seeded in the upper insert and co-cultured with decidual cells for 24–48 h (Figure 5D). These experiments showed that, in combination with TGF-β1-activated decidual cells in co-culture can significantly enhance trophoblast invasion and migration, and the activation of this TGF-β1 can reverse the suppression of decidual cells by IGF2BP3 overexpression on the invasion and migration of their co-cultured trophoblast cells (Figures 5F–H).

### IGF2BP3 Increase the Resorption of the Abortion-Prone Mouse Model *via* Transforming Growth Factor Beta 1

**FIGURE 6:**
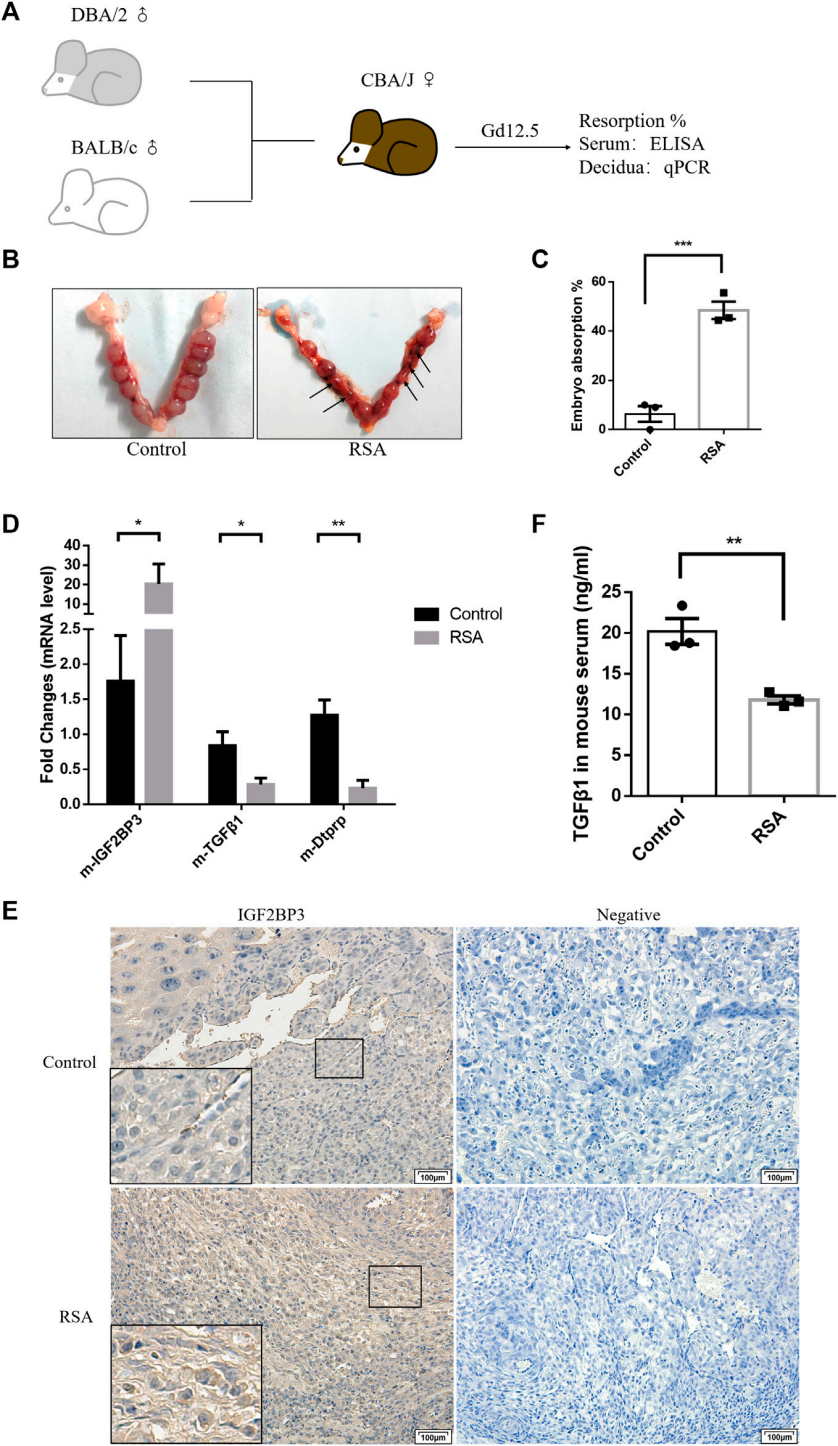
IGF2BP3 and TGF-β1 in the mouse abortion model **(A)** construction process of the mouse abortion model, **(B,C)** confirmed the success of the animal model by statistically analyzing the embryo resorption rate, **(D)** IGF2BP3, TGF-β1, and Dtprp mRNA levels in the decidual tissues of RSA mouse, and controls were measured by qPCR, **(E)** immunohistochemical staining showed the localization and relative quantification of IGF2BP3 in the decidual tissues of recurrent spontaneous abortion (RSA) and normal pregnancy (HC) model mice. Brown staining represents the target protein. Inserts show a higher magnification of the square area. Scale bar = 100 μm, and **(F)** expression levels of TGF-β1 in the serum of mice in RSA and control groups were determined by ELISA. *n* = 3 for each group. *p < 0.05, **p < 0.01, and ***p < 0.001 vs. control.

To validate the results of the aforementioned tissue and cell experiments, we investigated the effects of IGF2BP3 in a well-established mouse model for studying RSA (Figure 6A). We confirmed the success of the animal model by statistically analyzing the embryo resorption rate on day 12.5 of gestation between mice in the control and RSA model groups (Figures 6B,C).

We used qPCR analysis of first trimester decidual tissues from RSA and control mice to determine whether IGF2BP3 involve in the abortion-prone animal model. IGF2BP3 expression was considerably higher in the decidual tissues of RSA mice compared to controls at the mRNA level. (Figure 6D), thus indicating a possible damaging role of its excessive accumulation in the development and functional regulation of decidua. In contrast, the expression levels of TGF-β1 and the murine decidualization marker molecule Dtprp were significantly downregulated (Figure 6D). The immunohistochemistry results further validated the same decidual cell localization and expression levels of IGF2BP3 in RSA or control mice (Figure 6E). Meanwhile, we could observe fewer bulky discoid decidual cells and more spindle type endometrial stromal cells in the decidua of RSA mice, which was the same as described in Figure 2G (Figure 6E). These indicate that RSA model mice do present with impaired decidualization and that there is a syntenic correlation with TGF-β1.

To verify that IGF2BP3 is regulated by TGF-β1 regulation of the maternal–fetal interface cross talk also has effects in murine abortion models, we collected serum from RSA and control mice and measured TGF-β1 expression levels in them using an ELISA kit. It displayed that TGF-β1 levels were significantly reduced in the serum from RSA compared to the control group (Figure 6F). This suggests that IGF2BP3 may also regulate trophoblast invasion and migration by modulating cytokine communication (including TGF-β1) at the maternal–fetal interface in mouse models of miscarriage.

**FIGURE 7:**
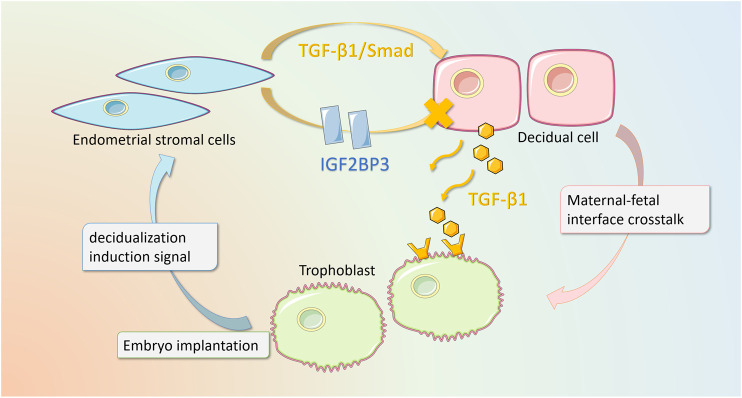
Schematic diagram of IGF2BP3 playing a regulatory role in decidualization and maternal–fetal interface cross talk. Our study elucidates that in recurrent pregnancy loss, the overexpression of IGF2BP3 in endometrial stromal cells leads to an impairment of decidualization through the inhibition of TGF-β1/Smad-signaling pathway. In addition, this mutation interferes with the cytokine cross talk between decidual cells and trophoblast cells, which in turn inhibits trophoblast migration and invasion.

In summary, IGF2BP3 overexpression regulates the TGF-β1-signaling pathway, impairing the cytokine cross talk between decidual cells and trophoblasts, which in turn inhibits trophoblast migration and invasion (Figure 7).

## Discussion

The risk of RSA has been demonstrated to be influenced by genetic anomalies, immunological dysfunction, structural abnormalities, endocrine abnormalities, infection, and environmental variables. However, the mechanisms underlying RSA occurrence remain elusive. Deficiency in decidualization has been widely recognized as one of the major cause of spontaneous abortion (Larsen et al., 2013). Decidualized HESCs can be used as the sensors of embryo quality upon implantation. Normal decidualization rejects poor-quality embryo implantation and causes rapid demise through menstruation-like shedding, which represents a natural embryo selection mechanism that restricts maternal investment in developmentally deficient pregnancies (Gellersen and Brosens, 2014). Abnormal decidualization reduces decidual receptivity, including limiting embryo implantation and failure to maintain pregnancy.

As an RNA binding protein, IGF2BP3 maintains mRNA stability and regulates its transcriptional expression by binding to the 5-untranslated region of mRNA-expressing IGF2 and activating transcriptional targets. IGF2BP3 has different targets in cell differentiation, proliferation, and other systems. It may regulate the biological behavior of decidualization and further proliferation and re-differentiation of HESCs to form decidual tissue through binding with other transcription factors or downstream pathways, and further affecting the occurrence and development of RSA (Haouzi et al., 2011).

In this study, we investigated the role of IGF2BP3 in the decidualization process and described its relationship with RSA. The expression of IGF2BP3 at both the mRNA and protein levels was significantly higher in the decidua of RSA patients than that of HC patients. Experiments in HESC *in vitro* showed that IGF2BP3-overexpressing cells had reduced the expression of IGFBP1 and PRL after decidualization induction and led to deficient morphological redifferentiation of human embryonic stem cells after decidualization. This may indicate that high IGF2BP3 expression reduces the decidualization capacity of HESCs and induces RSA. Interestingly, IGF2BP3 levels were not significantly downregulated during decidualization, and silencing IGF2BP3 did not result in increased decidualization. This may be related to the alternative functions of IGF2BP1 and IGF2BP2 and homologous molecules of IGF2BP3 (Huang et al., 2018). In the future, the roles of IGF2BP1 and IGF2BP2 in RSA deserve further study, which may contribute to deeply clarify the comprehensive mechanism of RSA development.

In addition, we also found that IGF2BP3 overexpression was accompanied by the decreased expression of TGF-β and MMP9. Meanwhile, the expression of TGF-β and MMP9 was also significantly elevated during decidualization. It has been shown that TGF-β is an important cytokine in decidualization, and silencing its expression can lead to impaired decidualization and induce the occurrence of RSA. In our study, we demonstrate that TGF-β1 and MMP9 are expressed at reduced levels in the decidua of RSA patients. Moreover, IGF2BP3 inhibits TGF-β1 activation and expression to impaired decidualization of HESCs, which may be mediated by the TGF-β/Smad pathway as our previous article (Chen et al., 2013). More importantly, the TGF-β1 supplementation can increase their expression levels of IGFBP1 and PRL and improve their impaired decidualization levels in HESC cells with IGF2BP3 overexpression, which further demonstrate the pivotal role of IGF2BP3 indcued the impairment of decidualization.

Emerging evidence indicate that endometrial stromal cells undergo cell cycle exit after embryo implantation, differentiating into decidual cells with secretory functions. Arresting cell cycle exit leads to abnormal decidualization. Cell cycle exit and differentiation are required for proper decidualization (Wang et al., 2018). At the G1 to S and G2 to m checkpoints, the cell cycle is precisely controlled. Most differentiated cells are arrested at the G1-to-S checkpoint, that is, at the G0/G1 phase, unable to enter S-phase and hence inhibit proliferation. Our Study showed that after 2–4 days of camp plus MPa treatment, the percentage of ESC cells in G0/G1 phase increased, and the percentage of cells in S-phase decreased, indicating that the cell cycle was stopped in the G0/G1 phase.

The present experiment used HESC cells with knockdown or overexpression of IGF2BP3, which were examined by flow cytometry for cell cycle changes during decidualization differentiation. We imprinted the induced shortening of S-phase of cells *in vitro*, which was the same as most findings (Logan et al., 2012). In turn, we demonstrated that HESC cells-overexpressing IGF2BP3 induced S-phase accumulation, an inability to programmed exit from the cell cycle and differentiation into decidual cells during decidualization. However, knockdown of IGF2BP3 decreased S-phase accumulation and inhibited cell proliferation. This suggests that IGF2BP3 may play an important role in G1/G0 cell arrest, an important link, in the mechanism of the endometrial cell decidualization process.

One question naturally arises: how does IGF2BP3 inhibits ESCs from exiting the cell cycle and entering differentiation? Several studies have shown that TGF-β1 as TGF-β superfamily members, which mainly regulate cell differentiation and proliferation, negatively regulate CDK activity and play a crucial role in G0/G1 cell cycle arrest (Zhang et al., 2017). Therefore, we further examined the activation of TGF-β pathway effects on the cell cycle of IGF2BP3-overexpressing cells. The results showed that TGF-β supplementation may enable promotion of its exit from the cell cycle during decidualization, reducing the accumulation of cells in S-phase, that is, it is possible that the impaired decidualization resulting from IGF2BP3 overexpression is through mediating TGF-β1 arrests cell cycle exit, unable to differentiate into decidual cells, which in turn causes RSA.

Furthermore, cross talk *via* numerous paracrine and autocrine factors at the maternal–fetal interface with decidual cells, which produce a vast variety of cytokines and express proteins such as TGF-β1 and MMP9 that alter trophoblast invasiveness, delicately regulates trophoblast invasion in normal pregnancy. The present study found that TR8/SVneo cells, an extravillous trophoblast cell line treated with IGF2BP3-overexpressing cell culture supernatant, had worse scratch-healing ability, and IGF2BP3 knockout cell culture supernatant promoted the migration of TR8/SVneo cells. The same results were confirmed in the co-culture system, in which both the migration and invasion abilities of TR8/SVneo co-cultured with IGF2BP3-overexpressing cells were reduced compared to their respective controls, whereas the migration and invasion abilities of the knockdown group were significantly increased.

Since earlier experiments has demonstrated that the TGF-β1 expression level is regulated by IGF2BP3, we sought to explore whether this regulation occurs similarly for exocrine TGF-β message interactions and trophoblast invasion at the maternal–fetal interface cross talk. Subsequently, we found that IGF2BP3 knockout cells secreted more TGF-β1 in the culture supernatant of HESC cells decidualization, whereas the overexpression of IGF2BP3-suppressed TGF-β1 secretion. The adding of exogenous TGF-β1 supplementation, the co-culture system, and wound healing assay also supported the conclusion that the overexpression of IGF2BP3 in decidual cells may inhibit the invasion-related molecule TGF-β at the maternal–fetal interface through aberrant blockade of the TGF-β1 expression, thereby hindering trophoblast invasion and leading to adverse pregnancy outcomes. SGN reports have shown that IGF2BP3 acts as an m6A methylation reader and negatively regulates TGF-β, possibly by regulating the promoter methylation of the TGF-β/R1-Smad2 pathway (Li et al., 2021b). But whether this pathway is regulated and bound specifically by which transcription factors and is associated with the mRNA structure stabilizing ability of IGF2BP3 awaits further investigation. Finally, we successfully established the RSA animal model, and found that the TGF-β1 level in the serum of RSA is lower than that of normal pregnant mouse.

## Conclusion

In conclusion, our investigation found the elevated IGF2BP3 expression in decidual tissue of RSA patients. According to the *in vitro* experiments and animal experiments, we demonstrated that IGF2BP3 plays a key regulatory role in decidualization of the human endometrium, and the overexpression of IGF2BP3 mediates the dysfunction of TGF-β1 resulted in impaired decidualization and decreased function of trophoblast invasion due to abnormal cross talk at the maternal–fetal interface, predisposing women to RSA. Our demonstration of a functional link among IGF2BP3, human decidualization, and RSA sheds new light on the mechanistic role of IGF2BP3 in the pathogenesis of RSA, and the underlying study of cytokine information communication at the maternal–fetal interface in pregnancy. In addition, our work suggests a potential IGF2BP3-based therapeutic strategy for the management of RSA. Additional studies are warranted to evaluate additional functions disrupted by IGF2BP3 dysregulation.

## Data Availability

The original contributions presented in the study are included in the article/Supplementary Material, further inquiries can be directed to the corresponding authors.

## Abbreviations

RSA, recurrent spontaneous abortion; IGF2BP3, insulin-like growth factor II mRNA-binding protein 3; TGF-β1, transforming growth factor β1; hESCs, human endometrial stromal cells; c-AMP, cyclic adenosine monophosphate; and IGF2, insulin-like growth factor 2.
