# Supplementary material for: The Mechanism of Insulin-Like Growth Factor II mRNA-Binging Protein 3 Induce Decidualization and Maternal-Fetal Interface Cross Talk by TGF-β1 in Recurrent Spontaneous Abortion
Source: Front Cell Dev Biol. 2022 Apr 8;10:862180. doi: 10.3389/fcell.2022.862180 (PMC9023862; doi:10.3389/fcell.2022.862180)
Supplement: Supplementary file 1 [file Table1.DOCX]

**Supplementary table 1**

| Primers | Sequence | |
| --- | --- | --- |
| h-IGF2BP3 | F | GTCAAGTGCAGAAGTTGTTGTC |
|  | R | GCAATCTGTCTTTGGTTTGGC |
| h-IGFBP1 | F | CTATGATGGCTCGAAGGCTC |
|  | R | TTCTTGTTGCAGTTTGGCAG |
| h-PRL | F | CTACATCCATAACCTCTCCTCA |
|  | R | GGGCTTGCTCCTTGTCTTC |
| h-TGF-β1 | F | GTTGAGCCGTGGAGGGGAAA |
|  | R | GGCCATGAGAAGCAGGAAAG |
| h-MMP9 | F | GTCCACCCTTGTGCTCTTCC |
|  | R | CGACTCTCCACGCATCTCTG |
| h-GAPDH | F | GGAGTCCACTGGCGTCTTCA |
|  | R | GTCATGAGTCCTTCCACGATACC |
| m-TGF-β1 | F | CTCCCGTGGCTTCTAGTGC |
|  | R | GCCTTAGTTTGGACAGGATCTG |
| m-Dtprp | F | AGCCAGAAATCACTGCCACT |
|  | R | TGATCCATGCACCCATAAAA |
| m-IGF2BP3 | F | CCCTTCAACCCTGACTCCT |
|  | R | CCATCCGCACTTTAGCATC |
| m-GAPDH | F | TCGCTCCTGGAAGATGGTGAT |
|  | R | CAGTGGCAAAGTGGAGATTGTTG |

**Table 1**: Primers Sequence.
