# Supplementary material for: The Mechanism of Insulin-Like Growth Factor II mRNA-Binging Protein 3 Induce Decidualization and Maternal-Fetal Interface Cross Talk by TGF-β1 in Recurrent Spontaneous Abortion
Source: Front Cell Dev Biol. 2022 Apr 8;10:862180. doi: 10.3389/fcell.2022.862180 (PMC9023862; doi:10.3389/fcell.2022.862180)
Supplement: Supplementary file 2 [file Table2.DOCX]

<https://www.jianguoyun.com/p/DYpOZ6QQutubChin0aoE>

<https://www.jianguoyun.com/p/DcD_UsYQytGbChio1qgE>

<https://www.jianguoyun.com/p/DcNDvLUQytGbChim1qgE>

https://www.jianguoyun.com/p/DdF9kcMQytGbChjej7AE
